# Mutational Patterns in RNA Secondary Structure Evolution Examined in Three RNA Families

**DOI:** 10.1371/journal.pone.0020484

**Published:** 2011-06-17

**Authors:** Anuj Srivastava, Liming Cai, Jan Mrázek, Russell L. Malmberg

**Affiliations:** 1 Institute of Bioinformatics, University of Georgia, Athens, Georgia, United States of America; 2 Department of Computer Science, University of Georgia, Athens, Georgia, United States of America; 3 Department of Microbiology, University of Georgia, Athens, Georgia, United States of America; 4 Department of Plant Biology, University of Georgia, Athens, Georgia, United States of America; University of Wyoming, United States of America

## Abstract

The goal of this work was to study mutational patterns in the evolution of RNA secondary structure. We analyzed bacterial tmRNA, RNaseP and eukaryotic telomerase RNA secondary structures, mapping structural variability onto phylogenetic trees constructed primarily from rRNA sequences. We found that secondary structures evolve both by whole stem insertion/deletion, and by mutations that create or disrupt stem base pairing. We analyzed the evolution of stem lengths and constructed substitution matrices describing the changes responsible for the variation in the RNA stem length. In addition, we used principal component analysis of the stem length data to determine the most variable stems in different families of RNA. This data provides new insights into the evolution of RNA secondary structures and patterns of variation in the lengths of double helical regions of RNA molecules. Our findings will facilitate design of improved mutational models for RNA structure evolution.

## Introduction

Molecules of RNA perform biological functions which require that they fold into specific secondary and tertiary structures. Conservation of these structures may be as important as, or more important than, sequence conservation during the course of RNA evolution [1], [2]. The associated base pairing in the double helical region of the RNA molecules is retained via patterns of compensatory mutations across sequences (covariation). Comparative methods for the determination of RNA secondary structures rely on detecting these compensatory mutations [3], [4].

Although many structural elements (stem-loops, pseudoknots) are conserved within a given RNA family, there is also variation in the presence or absence of certain stem-loops and pseudoknots across evolution, and there is variation in the length of corresponding double-helical regions [5], [6], [7], [8]. The types of variation that might be observable when comparing RNAs thus include single base substitutions, insertions and deletions, base-pair substitutions and insertions and deletions within a conserved stem, and insertion and deletion of entire secondary structure elements.

The patterns of RNA base and base pair changes have been both studied and modeled. One of the earliest models was developed by Knudsen et al. [9]; it incorporates the information of evolutionary history during RNA secondary structure prediction. Other studies analyzed patterns of compensatory mutations in RNA evolution [10] and showed the existence of variable rates of evolution across different rRNA structural elements [11]. A comparison of various mutational models describing the evolution of RNA secondary structure is presented by Savill et al. [12]. The patterns of compensatory mutations in RNA structures have been summarized in a matrix called RIBOSUM by analogy with the BLOSUM series of protein matrices; this matrix was developed and used in the RNA search program *RSEARCH* [13].

Recently, evolutionary models that address structural variation have been proposed. Holmes [14] developed a model of RNA structure evolution, which incorporates insertions and deletions of bases, base pairs, and whole stems. This model was based on the TKF91 model of sequence evolution [15], [16]. Other recent models of RNA evolution include the non-reversible generative (birth-death) evolutionary model for insertions and deletions [17], and the evolutionary triplet model based on a transducer composition algorithm [18]. One important potential application of the evolutionary triplet model is the inference of ancestral sequences for a set of diverged RNAs.

Our primary goal in this study was to determine the evolutionary and mutational patterns in double helical regions of RNA secondary structures that are responsible for variability in stem length, focusing on those that lead to stem-insertion and deletion. We chose to work with tmRNA (found in bacteria and organelles), RNaseP A (bacterial), RNaseP B (bacterial) and eukaryotic telomerase RNA sequences. This selection was motivated by the availability of large, well annotated databases for these RNA sequences and structures [19], [20], [21], [22]. We mapped structural changes onto phylogenetic trees which were constructed from data independent of the tmRNA, RNaseP and telomerase RNA sequences. Mutational patterns, obtained from correlated evolution of paired bases within the same stem among the related species, were documented by creating single and double nucleotide substitution matrices. In addition to determining the mutational patterns that lead to variability within individual stems, we also examined variability attributed to each stem by principal component analyses (PCA) of the stem length data. Our results build-on and extend early analyses of RNA secondary structure for tmRNA [8], [23], RNaseP [6], [24], [25] and telomerase RNA [5], [7], [26], [27].

## Materials and Methods

### 1. Alignment analysis

We obtained structural alignments for tmRNAs and RNasePs from the tmRNA database [20] and the Ribonuclease P database [19], respectively. Vertebrate, Ciliate and *Saccharomyces*, *Kluyveromyces* telomerase RNA structural alignments were obtained from Rfam [21] and the telomerase database [22], respectively. We preferred these databases over Rfam, as we believed that these databases are specialized for particular molecules and therefore contain better quality structural alignment; they provided expert annotation of the various structures (stem-loops, pseudoknots) across the sequence alignments. The alignments consisted of 268, 126, 25, 35, 22, 7 and 6 sequences for the tmRNA, RNaseP A, RNaseP B, and the Vertebrate, Ciliate, *Saccharomyces* and the *Kluyveromyces* telomerase RNAs, respectively. We chose *K. lactis* structure as a consensus for all 6 species of *Kluyveromyces*, as the telomerase database contains the annotation for the conserved segments only and Rfam has the alignment only for *Saccharomyces* species. Therefore, we used the *K. lactis* structure as a consensus and predicted additional helices in the segments which are unique to other *Kluyveromyces* species using *RNAfold* at default parameters (ViennaRNA-1.8.4) [28], [29], [30].

Except for RNaseP A and RNaseP B, the numbers of sequences used in our study are greater than or equal to the number of sequences present in the seed alignment of the Rfam database. We excluded RNaseP A and RNaseP B sequences that did not have corresponding rRNA sequences in the ribosomal database project [31]. *RNApasta* [32] was used to determine the length of each stem and loop and the stems involved in the RNA pseudoknot formation. This program takes predetermined RNA structural alignment as input and outputs the length of each stem and loop and information about the stems involved in the pseudoknot formation for each RNA molecule. All the alignments used in study along with their secondary structure model were included in the supplementary material (Text S1, Text S2, Text S3, Text S4, Text S5, Text S6, Text S7).

### 2. Phylogenetic analysis

We obtained rRNA sequences for the same species whose sequences were in the tmRNA and RNaseP datasets from the Ribosomal Database Project [31]. For the Ciliate and *Kluyveromyces* telomerase RNAs, corresponding rRNA sequences were obtained from the comparative rRNA website [33]. These rRNA sequences were used to create a reference phylogenic tree on which structural characters for each family of RNA were mapped. The vertebrate reference tree was obtained from the tree of life project [34] and final branches were adjusted manually from tree created by using the cytochrome B protein sequences. The accession number of cytochrome B sequences obtained from Swiss-Prot is given in supplementary Table S1. For *Saccharomyces*, the reference tree was obtained from the *Saccharomyces* phylogeny website (http://www.genetics.wustl.edu/saccharomycesgenomes/yeast_phylogeny.html).

The reference phylogenetic trees were built by *MrBayes3.1.2* program [35], [36]. The details of all the *MrBayes* parameters is given in supplementary Table S2 and the reference tree for each family of RNA under study is shown in the supplementary Figure S1.

We used the *Mesquite (version 2.74 (build 550))* program [37] to map the tmRNA, RNaseP and telomerase RNA stem lengths onto the reference phylogenetic tree. The history of each character (the stems) was traced onto the tree using the “reconstruct ancestral state” module of *Mesquite* with maximum parsimony. Given the tree and observed character distribution, this method finds the ancestral states that minimize the number of steps of character change. The cost of change for the continuous data from state x to state y is (x–y) which can be linear or squared; we used the default squared method as it can handle the trees with polytomies. *Dnapars(version 3.5c)* [38], a DNA parsimony program in the *Phylip* suite, was used to construct the hypothetical ancestral sequence at each node of the tree. This program counts the number of changes of bases needed on a given tree. We generated the hypothetical ancestral sequences by turning on the user tree and printing the sequences at the node of the tree options.

### 3. Structure evolution analysis

We manually determined which stems were variable across the phylogenetic tree; if one of the branches at the nodes containing closely related species were variable with respect to stem-loops then all the RNA sequences belonging to that particular node were used in the further analysis. We collected the underlying sequences for those stems from our alignment file. Afterwards, we created two types of base pair substitution matrices for each type of RNA under study. The base pair substitution matrices summarize information about the mutations that affect the pairing ability of the RNA molecules. The first matrix was created by counting the base changes that occur in the stem regions of extant sequences (those at the leaves of the trees). The second matrix was created by comparing the changes that occurred with respect to reconstructed ancestral sequences present at the corresponding node in the tree. Similarly, we also created two single nucleotide substitution matrices.

We transformed the counts in each cell of the matrices into observed/expected values using the formula: *A_ijkl_* = log_2_ ((*f_ijkl_*)/(*f_ij_f_kl_*)) where *A_ijkl_* is the value in any cell of the matrix, *f_ijkl_* is the frequency of base pair change for that cell, *f_ij_* and *f_kl_* are frequency of individual base pair involved in that change. Similarly, in the single nucleotide substitution matrix, observed/expected values were calculated by the formula: *A_ij_* = log_2_ ((*f_ij_*)/(*f_i_ f_j_*)) where *A_ij_* is the value in any cell of the matrix, *f_ij_* is the frequency of single nucleotide change for that cell, *f_i_* and *f_j_* are frequency of single nucleotide involved in that change. The expected values were calculated by obtaining the frequencies of nucleotides/base pairs within the matrices.

We also performed principal components analysis (PCA) on the stem length data obtained from the *RNApasta* program. Prior to performing PCA, we clustered data by k-means clustering [39] and then used the PCA to display the clusters. K-means clustering assigns each object (RNA molecule) into a predefined number (*k*) of clusters; we grouped the RNA molecules from different species based on similarity in their stem lengths. Both of the above analyses were performed using the R (R 2.9.1) statistical programming language.

## Results

### 1. Variable and conserved regions

**Figure 1:**
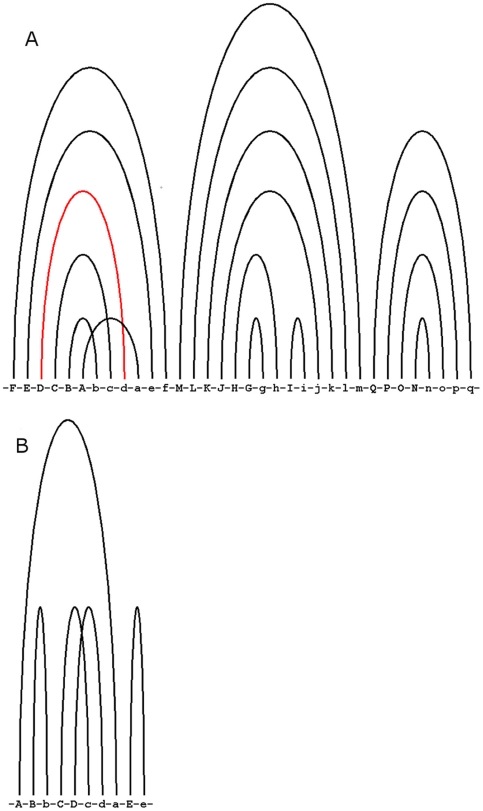
RNApasta arc diagram showing the ancestral state of each stem. RNA secondary structure diagram labeled with *RNApasta* annotation showing the ancestral state of each stem in terms of presence/absence of it, for A) Vertebrate telomerase RNA B) Ciliate telomerase RNA; the black and red of the each stem indicates the presence and absence, respectively. A crossing pattern of arcs indicates a pseudoknot. Each alphabet in the figure represents an RNA stem (*RNApasta* notation). [also see supplement figure S3 for other RNA families].

We used arc diagrams (Figure S2) generated by *RNApasta* [32] to display the length variability shown by each stem for all lineages in three families of RNAs. In these figures stems are divided into three categories based on their variability and colored differently. In addition, based upon the results obtained from the “reconstruct ancestral state” module of *mesquite*, we showed the ancestral state of each stem in terms of the presence or absence of it at the root node using these arc diagrams (Figure 1 and Figure S3).

### 2. Types of changes in helical regions

We found that there are two kinds of changes which lead to variability in the presence or absence of specific stems. They are whole stem insertion/deletion and stem gain/loss due to base substitution/indels which create or disrupt secondary structure base pairs. A summary for the two types of changes for every stem in each family of RNAs is shown in Table S3. Among the more than 100 examples of stem-loop evolution listed, we selected several examples of two kinds of changes to discuss in detail.

#### 2.1 Whole stem insertion/deletion

**Figure 2:**
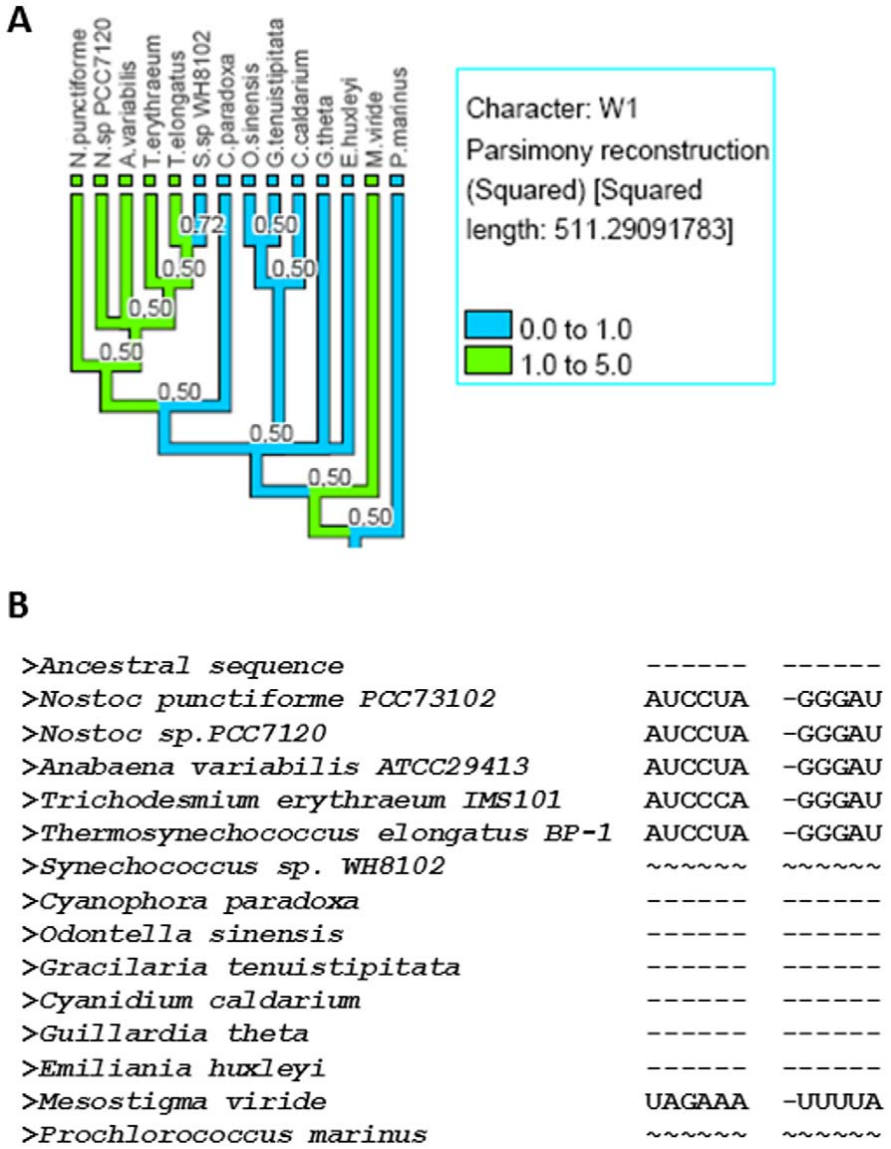
Cyanobacteria and chloroplasts' tmRNA stem W1 length mapped on rRNA phylogenetic tree. A) rRNA phylogenetic tree for cyanobacteria and chloroplasts' for the sequences of tmRNA under study with tmRNA stem W1 length values mapped on the rRNA tree; *MrBayes* calculated posterior probabilities of partition shown on each node of the tree and every branch is colored according to its stem length. The side bar shows the color legend for stem length values mapped onto the tree by *mesquite* using the parsimony ancestral reconstruction method. B) The tmRNA sequences including the reconstructed ancestral sequence (at the top generated by *Dnapars*) for the species present on the rRNA tree in the figure 2A are shown here. The ‘-’ and ‘∼’ indicate sequence absence and non-sequenced regions, respectively.

The first example is stem W1 of tmRNA, which is typically six base pairs long; it is involved in formation of an RNA pseudoknot (PK4) in cyanobacteria and chloroplasts' tmRNA. In cyanobacteria, this pseudoknot divides into two small pseudoknots PK4A and PK4B [23]. When we mapped stem W1 onto the tree (Figure 2A), we found that out of 14 related species, six species have this stem and out of seven cyanobacterial species, stem W1 is present in five of them. The presence/absence of structure is not certain for *Prochlorococcus marinus* and *Synechococcus* sp. *WH8102*, as this particular region is not sequenced. Interestingly, *Mesostigma viride* (fresh water algae) chloroplasts have this stem. *M. viride* represents the earliest diverging green plant lineage [40] and its chloroplast retains this stem which was lost in the other species' chloroplast tmRNAs. In order to determine whether this is an example of a stem insertion or deletion, we examined the reconstructed ancestral sequence at the common node (ignoring the *Prochlorococcus* and *Synechococcus* sequence during structure reconstruction) of RNA molecules of all these species. The alignment (Figure 2B) clearly suggests that this is an event of whole stem insertion as there is no sequence present at the ancestral node.

**Figure 3:**
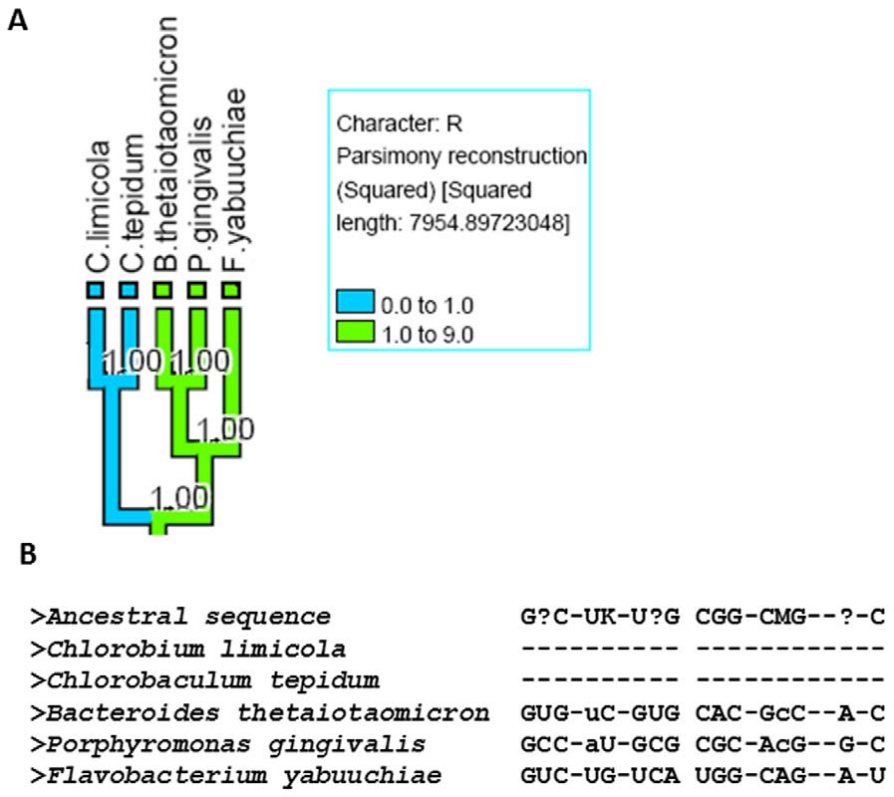
Bacteroidetes and Chlorobi RNaseP A stem R length mapped on rRNA phylogenetic tree. A) rRNA phylogenetic tree for Bacteroidetes and Chlorobi for the sequences of RNaseP A under study; RNaseP A stem R values mapped onto the rRNA tree; other legend are similar as figure 2A. B) The RNaseP A sequences including the hypothetical ancestral sequence (at the top generated by *Dnapars*) for the species present on the rRNA tree in the figure 3A are shown here. The ‘?’ indicates that the ancestral base is not certain at that position; other alphabet notation follows the standard IUPAC nucleotide code.

The second example is stem R of RNaseP A which is typically 10–12 base pairs long including the bulges. From the mapping of this stem onto the tree (Figure 3A), we found that this stem is present in full length in *B. thetaiotaomicron*, *P. gingivalis*, *F. yabuuchiae* and completely absent in *C. limicola* and *C. tepidum*. These species belong to Bacteroidetes/Chlorobi group. A reconstruction of the ancestral sequence (Figure 3B) suggests that this is an event of stem deletion in several derived sequences as there is sequence present at the ancestral node.

#### 2.2 Stem gain/loss due to base substitutions/indels

**Figure 4:**
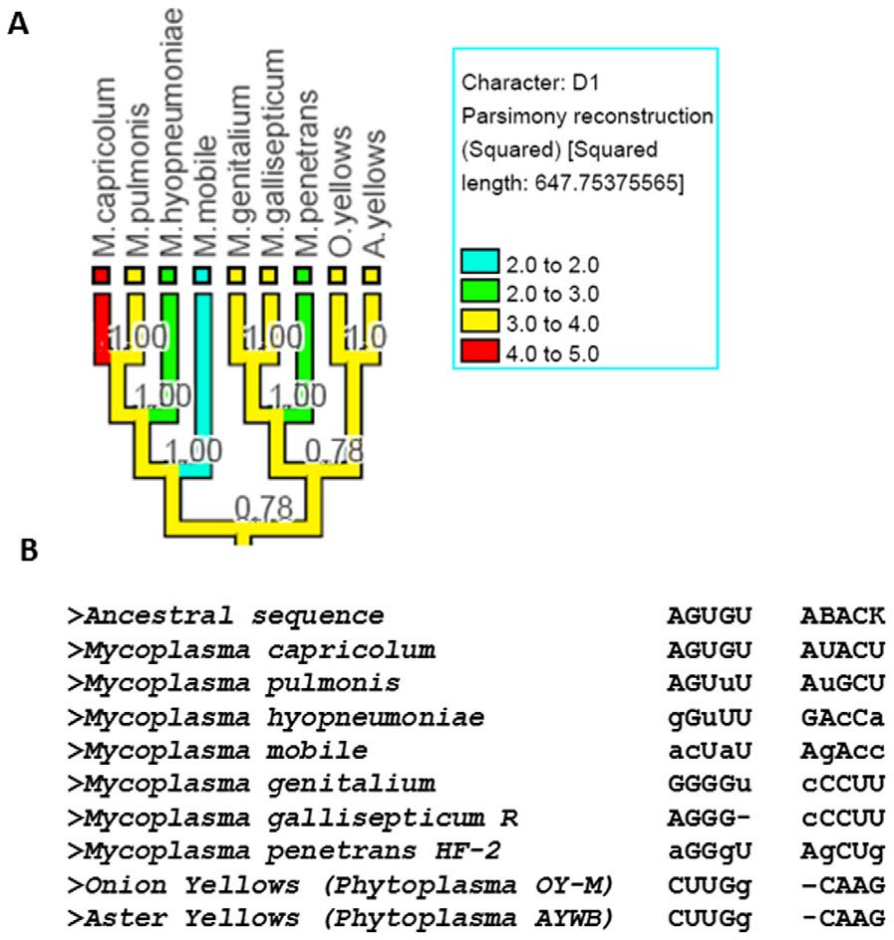
Mycoplasma tmRNA stem D1 length mapped on rRNA phylogenetic tree. A) rRNA phylogenetic tree for *Mycoplasma* for the sequences of tmRNA under study; tmRNA stem D1 values mapped on the rRNA tree; other legend symbols are similar to figure 2A. B) Underlying sequences of the species present in the tree shown in figure 4A; the small letter in the sequences indicate those bases which are mutated in such a way that they are not able to pair any more. The ‘-’ indicates the absence of base.

The variability in RNA secondary structure length also occurs due to mutations that create or eliminate base pairs in a stem region. These kind of mutations involves indels and substitutions. Two examples of stem gain/loss due to changes in base pairing potential are described below:

Stem D1 of tmRNA is up to 5 base pairs long. When we mapped the variation in this stem onto the tree (Figure 4A), we found that the size of the stem varies among members of the genus *Mycoplasma*. We then analyzed the underlying sequences (Figure 4B) and found that the nucleotides are present for all these species in the double-helical regions but they are mutating in certain positions in such a way that they are no longer able to pair, leading to a variable length for this stem in some tmRNA molecules.

**Figure 5:**
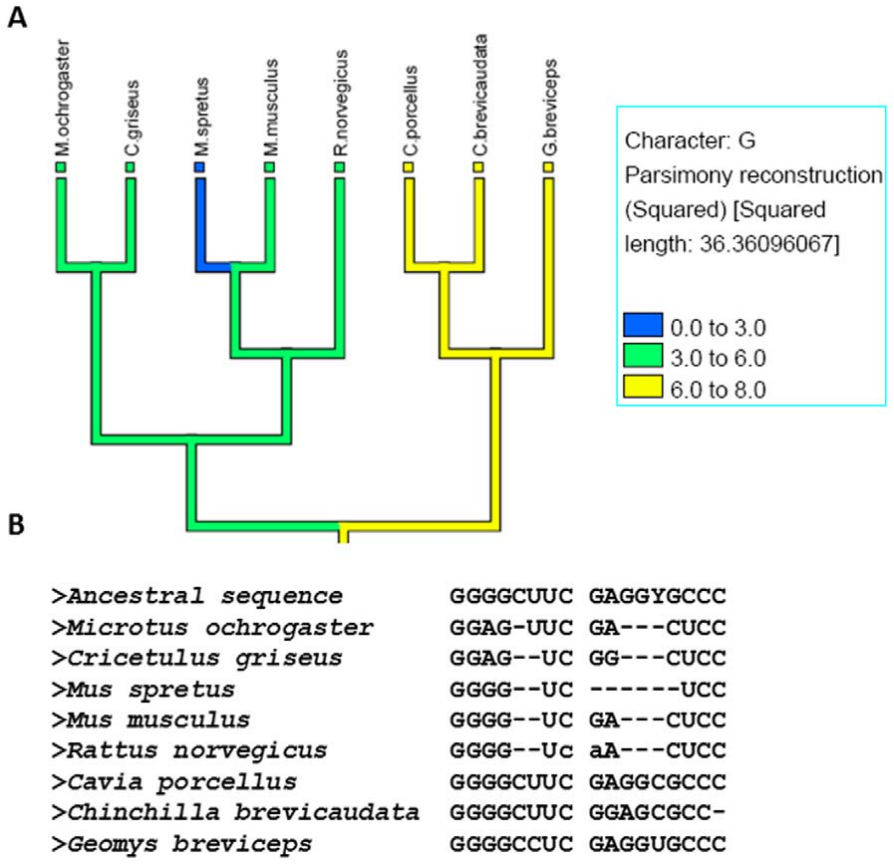
Vertebrates telomerase RNA stem G length mapped on reference tree. A) Reference phylogenetic tree for Vertebrates for the sequences of telomerase RNA under study; telomerase stem G values mapped on the reference tree; other legend symbols are similar as figure 2A. B) Underlying sequences of the species present in the tree shown in figure 5A; The ‘-’ indicates the absence of base.

Stem G of Vertebrate telomerase RNA is typically 8 base pair long. Mapping of stem length on a tree (Figure 5A) shows that this stem is variable among the species of order *Rodentia*. This stem is present in full length in *C.porcellus* and partially lost in other species. From the analysis of underlying sequences (Figure 5B), we found that this is an event of stem loss primarily due to base indels.

### 3. Substitutions associated with structural variation

We created base pair substitution matrices (Table S4 and Table S5) and single base substitution matrices (Table 1 and Table 2) combining the mutations from all three RNA families. These matrices were created by observing the variability in the size of each stem among RNA molecules of closely related species (Table 1 and Table S4) and variability with respect to hypothetical ancestral sequences (Table 2 and Table S5). The counts in each cell of the base pair matrix were transformed into observed/expected values. The total number of events scored in the base pair matrices constructed from extant/extant and ancestral/extant sequence comparisons are 53956 and 16903, respectively.

**Table 1:** Observed/expected value matrix combining the single nucleotide mutations from extant/extant sequences.

	A	C	G	U	-
**A**	4.17	-	-	-	-
**C**	2.41	3.36	-	-	-
**G**	2.44	1.25	2.05	-	-
**U**	2.07	1.79	0.63	1.43	-
**-**	0.29	−0.30	−0.98	−1.01	0.30

**Table 2:** Observed/expected value matrix combining the single nucleotide mutations from ancestral/extant sequences.

	A	C	G	U	-
**A**	2.98	−0.69	−0.16	−0.22	−3.61
**C**	−0.69	3.01	−1.14	0.20	−3.38
**G**	0.28	−0.47	2.44	−0.44	−3.23
**U**	−0.06	0.17	−1.07	2.76	−3.43
**-**	−1.21	−1.38	−1.61	−1.27	0.51

### 4. Principal component analysis on stem length data

We further analyzed the variation in stem lengths by k-means clustering [39] followed by principal component analysis (PCA). By comparing clustering results for different values of *k*, we determined that 5, 4, 3, 3, 3 were natural numbers of clusters for the sequences of tmRNA, RNaseP A, RNaseP B, Ciliate and Vertebrate telomerase RNA, respectively. The clustering followed the taxonomical classification of the species.

**Figure 6:**
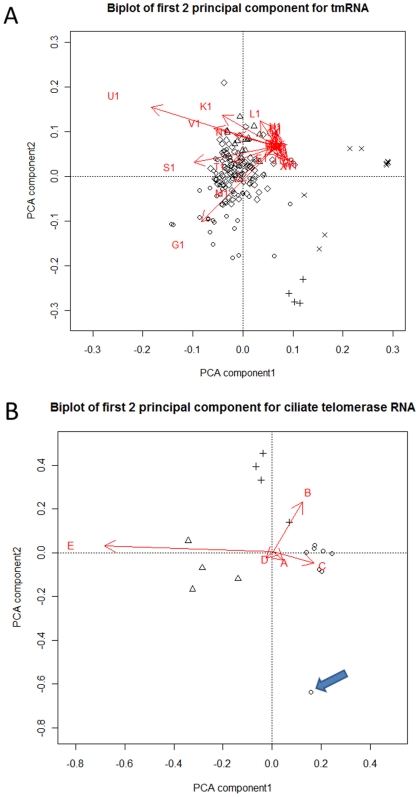
PCA Biplot for tmRNA and Ciliate telomerase RNA. Biplot of principal components for A) tmRNA B) Ciliate telomerase RNA; points in different shape represents clusters of species; partial tmRNA sequences were excluded from the analysis. Isolated species mentioned in the discussion are indicated by arrows on biplot.

We displayed the clusters on a PCA biplot to investigate further variance in stem lengths. The first 2 principal components explain 45% of the overall variance in stem lengths for tmRNA. The biplot of the first 2 principal components for tmRNA (Figure 6A) shows that stems U1 and G1 contribute most to the first and second principal components, respectively. For RNaseP A and RNaseP B, the first two components cover 78% and 80% of the variance, respectively. The biplot of the first 2 principal components for RNaseP A (Figure S4A) shows that the stems L and S contribute most to the first and second principal components, respectively. In fact, the vast majority of the stem length variance in the RNaseP A family can be attributed to these two stems. For the RNaseP B, the major contributors to the first and second principal components (Figure S4B) are stem C, K and Q, respectively. In the eukaryotic ciliate and vertebrate telomerase RNA, the first two components cover 95% and 80% of the variance, respectively. In the Vertebrates, stem F, D (Figure S4C) and in Ciliates stem E, B (Figure 6B) contribute most to the first and second principal components, respectively. We were not able to perform the PCA on *Saccharomyces* and *Kluyveromyces* stem length data as the number of sequences was fewer than number of dimensions (stems). For prokaryotic tmRNA and RNaseP, we investigated possible relationships of the first two principal components with biological properties of the organisms, including oxygen requirements, temperature, energy source and motility. However, we did not find any significant relationship between the biological properties and principal components. Detailed results of the clustering and symbols representing the species are presented in the Supplementary Table S6.

## Discussion

Our analysis of RNA secondary structures centers on documenting the mutational patterns responsible for the variation in the double helical regions, including insertion and deletions of whole stems as well as changes in the stem lengths. Our approach differs from previous studies of tmRNA [8], [23] and RNaseP ([6], [24], [25], [41], [42], [43] in using a reference phylogenetic tree on to which the stem characteristics of the respective RNAs are mapped (Figures 2, 3, 4, 5), as well as the other methods of data analysis, and in the number of sequences used. For telomerase RNA, comparative methods were previously used to help predict the consensus structures [5], [7], [26], [27], but there were no analyses of stem-loop evolution and the base pair changes that accompany it.

Based upon the variability obtained by mapping the structure characters onto the tree, we were able to determine the level of variability shown by every stem of each RNA family under study (Figure S1). We determined the relative frequency of the two categories of events responsible for the variation in the RNA secondary structure (Table 3). Our data suggests that models to describe RNA structure evolution have to consider both modes of stem appearance/disappearance; while stem insertion/deletion is the less common mode, the rates differs significantly among three RNA families (χ^2^ = 16.8019, df = 2, p-value = 0.0002247).

**Table 3:** Frequency of events in percentage responsible for variation in stem length in RNA secondary structure.

RNA family	Whole stem insertion/deletion(%)	Base substitution/indels(%)
tmRNA	43.6	56.4
RNaseP A	27.5	72.5
RNaseP B	15	85
Vertebrate telomerase RNA	9.4	90.6
Ciliate telomerase RNA	7.6	92.3
*Saccharomyces* telomerase RNA	11.2	88.8
*Kluyveromyces* telomerase RNA	43.4	56.6

We constructed matrices to summarize the changes in bases and base pairs that occurred in stems that were variable across the phylogenetic tree. Since we also reconstructed the ancestral sequences, we were able to compare ancestral sequences with extant sequences as well as extant sequences with each other. All the methods available for ancestral sequence reconstruction have their limitations [44], [45]; in particular parsimony and maximum likelihood may lead to sequences which contain fewer of the less common residues than they should [44], [45]. We chose parsimony for the sequence reconstruction since the ancestral RNA structure reconstruction was performed by parsimony, although both are based upon an underlying tree generated by Bayesian methods. The primary effect of the parsimony bias in ancestral sequence reconstruction on our results would be that the matrices comparing ancestral and current sequences would be conservative, slightly underestimating some of the rarer changes. The previously constructed RIBOSUM matrices [13] are based upon rRNA structure alignments, which are highly conserved molecules and therefore might not be suitable for the analyses, where the structure of the RNA is variable among the related species. In contrast, our matrices should be well-suited for such an analysis as they were derived from alignments showing structural variability in phylogenetically related species. Thus, we also have a gap column ‘-’ in the matrices showing the relative frequencies of indel events.

From the reconstructed ancestral state of each stem, obtained using mesquite, we found that in vertebrate telomerase RNA, stem D (Figure 1A) is absent from the root node. This stem is specific to mammals and is considered to be possibly involved in binding to the TERT protein [46]. The absence of this stem at the root node suggests that it has been acquired in the course of evolution and the lack of this stem in species other than mammals might indicate there is an alternative way to interact with TERT protein in these species.

We used principal components analysis to identify co-variable stems among the RNA molecules under study. The observation that stem U1 (involved in the formation of RNA pseudoknot PK4) is variable among the tmRNAs (Figure 6A) is consistent with our other observation that the PK4 pseudoknot is absent from chloroplasts and from some endosymbiont tmRNAs. Endosymbionts may be under relaxed selective pressure in order to maintain fast growth and therefore they may tolerate a less efficient stalled translation associated with a suboptimal tmRNA [47].

In the Ciliate PCA biplot (Figure 6B), we found that *Tetrahymena paravorax* separates from all other species. A comparison among the ciliate telomerase RNA sequences indicates that this is due to the absence of stem B in the *T. paravorax* telomerase RNA. In the previous studies of ciliate telomerase RNA, this helix has been suggested to be a primitive telomerase RNA structural feature and deletion of this stem in *T. paravorax* and in other hypotrich telomerase RNAs is considered to be example of convergent evolution [7]. Our ancestral arc diagram (Figure 1B) also showed the presence of this stem at the root node.

In summary, we implemented a new approach to analyzing RNA structure from an evolutionary prospective. From this analysis, we conclude that different types of mutations are responsible for the variation in the lengths of double helical regions of RNA. We documented the associated substitution patterns in log-odds matrices. We also demonstrate the usefulness of PCA in the analysis of the RNA structure alignment. PCA in combination with clustering can easily determine the outliers from the large structure alignment of RNA which can then be subjected to further analysis. Further studies like these of the evolutionary variability of RNA structure and the associated mutational patterns will be essential for improving computational programs that model RNA structures.

## Supporting Information

Figure S1 **Reference tree for all RNA families under study.** Reference tree for sequences of A) tmRNA B) RNaseP A C) RNaseP B D) Vertebrate E) Ciliate F) *Saccharomyces* G) *Kluyveromyces* telomerase RNA; node numbers are indicated in the circle on each tree.
(TIF)

Figure S2 **RNA secondary structure displaying stem variability drawn by RNApasta.** RNA secondary structure diagram labeled with *RNApasta* annotation for A) tmRNA B) RNaseP A C) RNaseP B D) Vertebrate telomerase RNA E) Ciliate telomerase RNA F) *Saccharomyces* telomerase RNA and G) *Kluyveromyces* telomerase RNA; the black, brown and red color of stems indicates that single length distribution is present in 71–100%, 41–70 and 1–40% of the species, respectively. The intersecting lines connecting two loop region indicates a pseudoknot. Each alphabet in the figure represents a RNA stem (*RNApasta* notation).
(TIF)

Figure S3 **RNApasta arc diagram showing ancestral state of each stem.** RNA secondary structure diagram labeled with *RNApasta* annotation showing the ancestral state of each stem in terms of presence/absence of it, for A) tmRNA B) RNaseP A C) RNaseP B D) *Saccharomyces* telomerase RNA and E) *Kluyveromyces* telomerase RNA; the black, red and brown color of the each stem indicates the presence, absence and ancestral state not resolved, respectively. A crossing pattern of arcs indicates a pseudoknot. Each alphabet in the figure represents a RNA stem (*RNApasta* notation).
(TIF)

Figure S4 **PCA Biplot for RNaseP A, RNaseP B and vertebrate telomerase RNA.** Biplot of principal components for A) RNaseP A B) RNaseP B and C) Vertebrate telomerase RNA; arrows followed by alphabet indicates RNA stems; points in different shape represents clusters of species; partial RNaseP A sequences were excluded from the analysis.
(TIF)

Table S1 **Accession numbers of cytochrome B sequences used in vertebrate reference tree creation.**
(DOC)

Table S2 **Detailed mcmc parameters used in** ***MrBayes*** **for reference tree creation.**
(DOC)

Table S3 **The process responsible for variation in the stem length.**
(DOC)

Table S4 **Observed/expected base pair substitution matrices combining the mutations among all three RNA families for the extant/extant sequence comparison.**
(DOC)

Table S5 **Observed/expected base pair substitution matrices combining the mutations among all three RNA families for the ancestral/extant sequence comparison.**
(DOC)

Table S6 **The species present in the each cluster in all studied RNA families in PCA.**
(DOC)

Text S1 **Alignment of tmRNA used in the study.**
(TXT)

Text S2 **Alignment of RNaseP A.**
(TXT)

Text S3 **Alignment of RNaseP B.**
(TXT)

Text S4 **Vertebrate telomerase RNA alignment.**
(TXT)

Text S5 **Ciliate telomerase RNA alignment.**
(TXT)

Text S6 ***Saccharomyces*** **telomerase RNA alignment.**
(TXT)

Text S7 ***Kluyveromyces*** **telomerase RNA alignment.**
(TXT)
