# Supplementary figures and images for: Mutational Patterns in RNA Secondary Structure Evolution Examined in Three RNA Families
Source: PLoS One. 2011 Jun 17;6(6):e20484. doi: 10.1371/journal.pone.0020484 (PMC3117835; doi:10.1371/journal.pone.0020484)

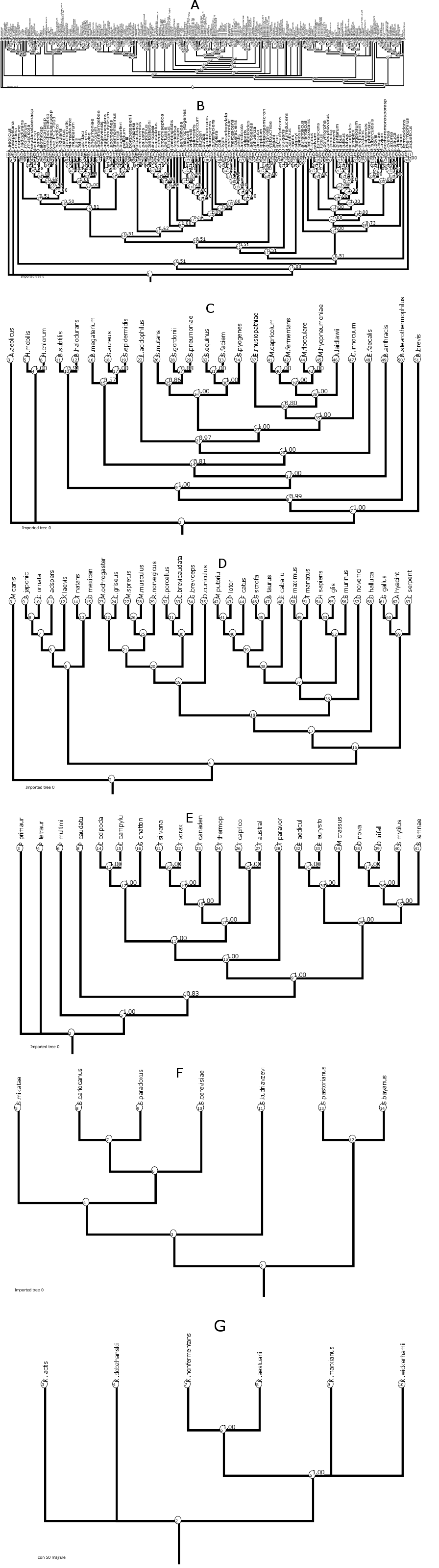

Supplement: Figure S1 — Reference tree for all RNA families under study. Reference tree for sequences of A) tmRNA B) RNaseP A C) RNaseP B D) Vertebrate E) Ciliate F) Saccharomyces G) Kluyveromyces telomerase RNA; node numbers are indicated in the circle on each tree. (TIF) [file pone.0020484.s001.tif]

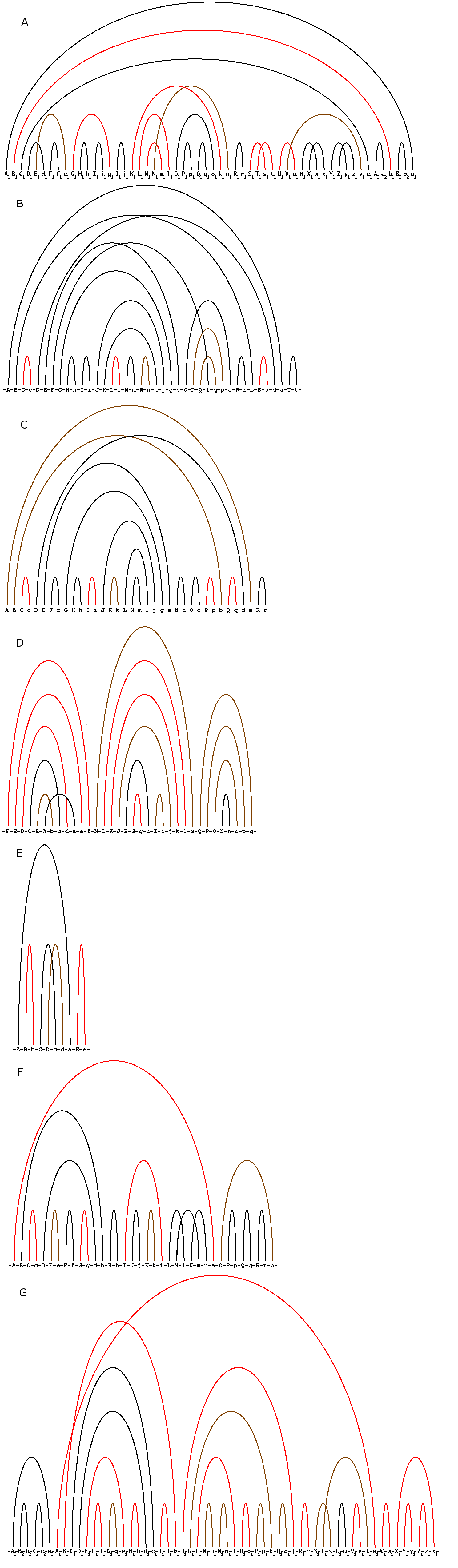

Supplement: Figure S2 — RNA secondary structure displaying stem variability drawn by RNApasta. RNA secondary structure diagram labeled with RNApasta annotation for A) tmRNA B) RNaseP A C) RNaseP B D) Vertebrate telomerase RNA E) Ciliate telomerase RNA F) Saccharomyces telomerase RNA and G) Kluyveromyces telomerase RNA; the black, brown and red color of stems indicates that single length distribution is present in 71–100%, 41–70 and 1–40% of the species, respectively. The intersecting lines connecting two loop region indicates a pseudoknot. Each alphabet in the figure represents a RNA stem (RNApasta notation). (TIF) [file pone.0020484.s002.tif]

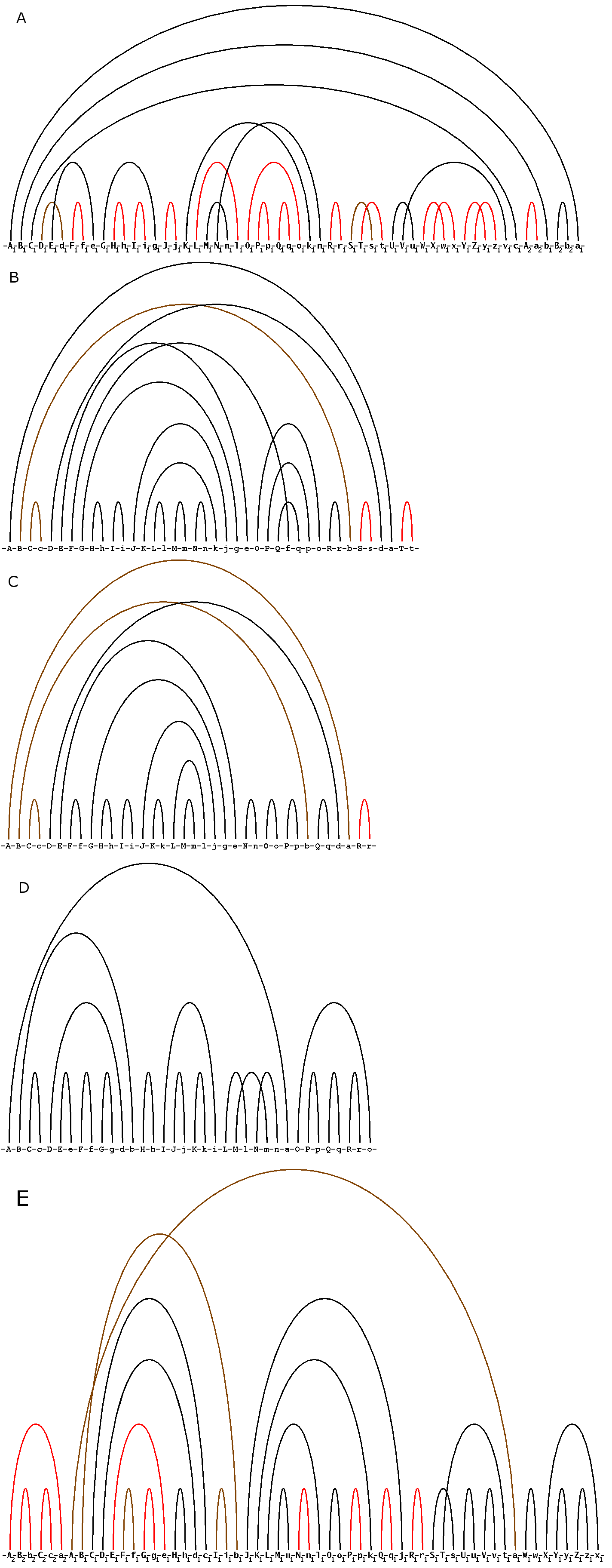

Supplement: Figure S3 — RNApasta arc diagram showing ancestral state of each stem. RNA secondary structure diagram labeled with RNApasta annotation showing the ancestral state of each stem in terms of presence/absence of it, for A) tmRNA B) RNaseP A C) RNaseP B D) Saccharomyces telomerase RNA and E) Kluyveromyces telomerase RNA; the black, red and brown color of the each stem indicates the presence, absence and ancestral state not resolved, respectively. A crossing pattern of arcs indicates a pseudoknot. Each alphabet in the figure represents a RNA stem (RNApasta notation). (TIF) [file pone.0020484.s003.tif]
