# Supplementary material for: Mutational Patterns in RNA Secondary Structure Evolution Examined in Three RNA Families
Source: PLoS One. 2011 Jun 17;6(6):e20484. doi: 10.1371/journal.pone.0020484 (PMC3117835; doi:10.1371/journal.pone.0020484)
Supplement: Table S1 — Accession numbers of cytochrome B sequences used in vertebrate reference tree creation. (DOC) [file pone.0020484.s005.doc]

| **Species** | **Accession** |
| --- | --- |
| *Mustelus canis* | Q1W9G9 |
| *Bufo japonicus* | A8CFB4 |
| *Ceratophrys ornata* | Q34193 |
| *Pyxicephalus adspersus* | B9VWL1 |
| *Xenopus laevis* | Q7YHP3 |
| *Typhlonectes natans* | Q9MI97 |
| *Dermophis mexicanus* | C9D8B9 |
| *Microtus orchrogaster* | A4K4X1 |
| *Cricetulus griseus* | B1PKV9 |
| *Mus spretus* | Q85QL4 |
| *Mus musculus* | Q401Y0 |
| *Rattus norvegicus* | A4UHZ2 |
| *Cavia porcellus* | Q6X9U4 |
| *Chinchilla brevicaudata* | Q8HBP8 |
| *Geomys breviceps* | Q5YJ89 |
| *Oryctolagus cuniculus* | Q9TEF8 |
| *Mustela putorius* | Q9MD71 |
| *Procyon lotor* | A4F2R1 |
| *Felis catus* | Q4LEU3 |
| *Sus scrofa* | Q9T567 |
| *Bos taurus* | Q5EG28 |
| *Equus caballus* | Q53YR5 |
| *Elephas maximus* | Q9XNF9 |
| *Trichechus manatus* | Q32QM3 |
| *Homo sapiens* | Q85BE0 |
| *Tupaia glis* | B6CUY7 |
| *Suncus murinus* | A9CDJ6 |
| *Dasypus novemcinctus* | Q8SDX7 |
| *Dasyurus hallucatus* | Q34321 |
| *Gallus gallus* | Q5D6Z9 |
| *Anodorhynchus hyacinthinus* | Q0ZSE1 |
| *Chelydra serpentina* | B5KFB4 |
