# Supplementary material for: Mutational Patterns in RNA Secondary Structure Evolution Examined in Three RNA Families
Source: PLoS One. 2011 Jun 17;6(6):e20484. doi: 10.1371/journal.pone.0020484 (PMC3117835; doi:10.1371/journal.pone.0020484)
Supplement: Table S2 — Detailed mcmc parameters used in MrBayes for reference tree creation. (DOC) [file pone.0020484.s006.doc]

|  | **nrun** | **ngen (million)** | **printfreq** | **Sample freq** | **nchains** | **burn-in** |
| --- | --- | --- | --- | --- | --- | --- |
| **tmRNA** | 2 | 5 | 1000 | 500 | 4 | 480 |
| **RNAseP A** | 2 | 50 | 20000 | 500 | 4 | 19520 |
| **RNAseP B** | 2 | 30 | 10000 | 500 | 4 | 6480 |
| **Vertebrate** | 2 | 30 | 10000 | 1000 | 4 | 90 |
| **Ciliate** | 2 | 1 | 10000 | 1000 | 4 | 50 |
| ***Kluyveromyces*** | 2 | 1 | 10000 | 100 | 4 | 50 |
