# Supplementary material for: Mutational Patterns in RNA Secondary Structure Evolution Examined in Three RNA Families
Source: PLoS One. 2011 Jun 17;6(6):e20484. doi: 10.1371/journal.pone.0020484 (PMC3117835; doi:10.1371/journal.pone.0020484)
Supplement: Table S3 — The process responsible for variation in the stem length. (DOC) [file pone.0020484.s007.doc]

Alphabet in the table indicates RNA stems and the number in the parenthesis indicates the node at reference tree where variability occurs. The reference tree for each family of RNA under study is shown in the figure S1.

**A**

| **tmRNA** | |
| --- | --- |
| **Stem gain/loss via whole stem insertion/deletion** | **Stem gain/loss via base substitutions/indels** |
| C1(12), E1(12), E1(421), G1(12), G1(365), H1(39), H1(89), H1(244), H1(365), I1(37), I1(244), I1(365), J1(174), J1(189), J1(198), J1(434), K1(12), K1(36),K1(157), K1(216), K1(424), L1(12), L1(157), L1(340), L1(424), L1(474), M1(12), M1(36), M1(340), N1(12), N1(157), O1(157), O1(164), P1(120), P1(135), P1(140), Q1(140), R1(12), S1(12), T1(16), T1(157), T1(189), U1(120), W1(12), X1(12), Y1(12), Z1(12), Z1(157) | B1(12), B1(36), B1(159), B1(174), B1(330), B1(345), B1(365), B1(378), B1(427), B1(445), C1(56), C1(157), C1(434), D1(12), D1(39), D1(59), D1(85), D1(365), D1(425), E1(148), E1(379), E1(434), G1(39), G1(135), G1(148), G1(157)¸ G1(174), G1(189), G1(434), G1(473), K1(189), L1(50), L1(120), M1(189), M1(236), M1(244), N1(189), O1(140), O1(148), P1(148), S1(36), S1(74), S1(120), S1(135), S1(340), S1(358), S1(378), S1(401), S1(424), S1(457), T1(36), T1(66), T1(120), T1(135), T1(340), T1(426), U1(36), U1(157), U1(244), U1(340), U1(426), V1(36), Z1(164) |

**B**

| **RNaseP A** | |
| --- | --- |
| **Stem gain/loss via whole stem insertion/deletion** | **Stem gain/loss via base substitutions/indels** |
| M(94), N(94), Q(205), R(160), S(17), S(84),S(94), S(211) | A(53), C(17), C(53), C(66), C(137), D(53), E(17), F(21), F(66), F(94), F(194), L(21), L(94), L(211), N(160), O(17), P(25), P(63), P(165), Q(17), S(231) |

**C**

| **RNaseP B** | |
| --- | --- |
| **Stem gain/loss via whole stem insertion/deletion** | **Stem gain/loss via base substitutions/indels** |
| K(39), M(39), Q(39) | A(39), C(15), C(24), C(39), I(10), I(15), I(24), I(39), K(10), K(15), P(4), P(10), P(15), P(24), P(39), Q(15), R(39) |

**D**

| **Vertebrate Telomerase RNA** | |
| --- | --- |
| **Stem gain/loss via whole stem insertion/deletion** | **Stem gain/loss via base substitutions/indels** |
| E(21), D(59), L(59), G(5) | F(5), F(30), F(38), F(49), F(52), E(5), E(52), B(21), B(30), B(52), A(21), M(5), M(21), M(30), M(52), L(5), K(5), K(19), K(36), K(59), J(5), J(21), J(59), G(19), G(38), G(52), Q(5), Q(30), Q(36), Q(59), P(5), P(19), P(36), O(5), O(19), O(36), O(59), N(19), N(36) |

**E**

| **Ciliate Telomerase RNA** | |
| --- | --- |
| **Stem gain/loss via whole stem insertion/deletion** | **Stem gain/loss via base substitutions/indels** |
| B(10) | A(30), B(2), B(30), C(12), C(10), D(2), D(12), D(10), D(30), E(2), E(30) E(35) |

**F**

| ***Saccharomyces* Telomerase RNA** | |
| --- | --- |
| **Stem gain/loss via whole stem insertion/deletion** | **Stem gain/loss via base substitutions/indels** |
| D(12), G(3) | A(3), B(3), C(3), D(3), E(3), E(12), F(3), G(12), I(3), I(12), J(3), K(3), L(12), O(3), P(3), Q(3), |

**G**

| ***Kluyveromyces* Telomerase RNA** | |
| --- | --- |
| **Stem gain/loss via whole stem insertion/deletion** | **Stem gain/loss via base substitutions/indels** |
| B2(2), C2(2), D2(2), A1(2), B1(2), E1(2), F1(2), G1(2), H1(2), Q1(2), R1(2), S1(2), V1(2), | C1(2), D1(2), I1(2), J1(2), K1(2), L1(2),M1(2), N1(2), O1(2), P1(2),T1(2), U1(2), W1(2), X1(2),Y1(2), Z1(2), A2(2) |
