# Supplementary material for: Mutational Patterns in RNA Secondary Structure Evolution Examined in Three RNA Families
Source: PLoS One. 2011 Jun 17;6(6):e20484. doi: 10.1371/journal.pone.0020484 (PMC3117835; doi:10.1371/journal.pone.0020484)
Supplement: Table S4 — Observed/expected base pair substitution matrices combining the mutations among all three RNA families for the extant/extant sequence comparison. (DOC) [file pone.0020484.s008.doc]

|  | **AA** | **AC** | **AG** | **AU** | **A-** | **CA** | **CC** | **CG** | **CU** | **C-** | **GA** | **GC** | **GG** | **GU** | **G-** | **UA** | **UC** | **UG** | **UU** | **U-** | **-A** | **-C** | **-G** | **-U** | **--** |
| --- | --- | --- | --- | --- | --- | --- | --- | --- | --- | --- | --- | --- | --- | --- | --- | --- | --- | --- | --- | --- | --- | --- | --- | --- | --- |
| **AA** | 8.84 | - | - | - | - | - | - | - | - | - | - | - | - | - | - | - | - | - | - | - | - | - | - | - | - |
| **AC** | 6.91 | 10.14 | - | - | - | - | - | - | - | - | - | - | - | - | - | - | - | - | - | - | - | - | - | - | - |
| **AG** | 5.69 | 5.88 | 7.23 | - | - | - | - | - | - | - | - | - | - | - | - | - | - | - | - | - | - | - | - | - | - |
| **AU** | 4.28 | 5.26 | 2.04 | 4.57 | - | - | - | - | - | - | - | - | - | - | - | - | - | - | - | - | - | - | - | - | - |
| **A-** | 4.38 | 5.09 | 2.43 | 2.32 | 7.00 | - | - | - | - | - | - | - | - | - | - | - | - | - | - | - | - | - | - | - | - |
| **CA** | 4.95 | 5.72 | 3.90 | 2.47 | 3.21 | 7.49 | - | - | - | - | - | - | - | - | - | - | - | - | - | - | - | - | - | - | - |
| **CC** | 4.53 | 6.74 | 3.21 | 2.80 | 3.63 | 4.43 | 7.64 | - | - | - | - | - | - | - | - | - | - | - | - | - | - | - | - | - | - |
| **CG** | 2.63 | 2.88 | 1.23 | 0.61 | -0.37 | 3.00 | 2.58 | 3.24 | - | - | - | - | - | - | - | - | - | - | - | - | - | - | - | - | - |
| **CU** | 3.35 | 5.84 | 1.89 | 2.09 | 1.48 | 3.89 | 5.48 | 1.45 | 5.85 | - | - | - | - | - | - | - | - | - | - | - | - | - | - | - | - |
| **C-** | 3.98 | 4.48 | 1.63 | 0.81 | 4.61 | 2.89 | 4.06 | 1.15 | 2.89 | 6.38 | - | - | - | - | - | - | - | - | - | - | - | - | - | - | - |
| **GA** | 4.35 | 4.60 | 2.06 | 1.42 | 1.63 | 3.14 | 2.32 | 0.07 | 1.36 | 0.19 | 5.56 | - | - | - | - | - | - | - | - | - | - | - | - | - | - |
| **GC** | 2.45 | 4.34 | 0.79 | 1.68 | -0.16 | 1.58 | 2.60 | -0.23 | 0.23 | 0.44 | 0.41 | 2.97 | - | - | - | - | - | - | - | - | - | - | - | - | - |
| **GG** | 4.21 | 4.91 | 3.69 | 1.09 | -0.37 | 4.05 | 2.67 | 0.48 | 2.48 | 0.48 | 2.51 | 0.79 | 5.38 | - | - | - | - | - | - | - | - | - | - | - | - |
| **GU** | 3.03 | 4.23 | 1.49 | 1.98 | 0.83 | 1.71 | 1.52 | -0.36 | 2.01 | 0.93 | 1.15 | 1.23 | 1.66 | 3.57 | - | - | - | - | - | - | - | - | - | - | - |
| **G-** | 2.86 | 4.58 | 0.99 | 1.11 | 4.37 | 2.28 | 0.99 | -0.80 | 1.25 | 4.13 | 1.23 | 0.32 | 1.21 | 1.41 | 5.65 | - | - | - | - | - | - | - | - | - | - |
| **UA** | 3.41 | 3.50 | 1.22 | 1.17 | 0.78 | 3.33 | 1.83 | 0.34 | 0.27 | 0.29 | 0.89 | -0.25 | 0.61 | 0.21 | -0.17 | 2.54 | - | - | - | - | - | - | - | - | - |
| **UC** | 3.49 | 4.99 | 1.82 | 1.55 | 1.14 | 2.99 | 3.49 | 0.05 | 2.99 | 0.89 | 1.29 | 0.80 | 1.49 | 0.32 | -0.08 | 1.21 | 3.64 | - | - | - | - | - | - | - | - |
| **UG** | 3.57 | 3.73 | 1.67 | 0.96 | -0.12 | 2.63 | 2.49 | 1.01 | 0.64 | 0.44 | 0.59 | -0.26 | 1.31 | -0.23 | -0.64 | 1.07 | 1.10 | 2.85 | - | - | - | - | - | - | - |
| **UU** | 3.80 | 4.80 | 1.57 | 1.82 | 0.73 | 3.06 | 2.97 | 0.00 | 2.92 | 0.89 | 1.06 | -0.05 | 1.47 | 0.92 | 0.51 | 1.04 | 2.03 | 1.15 | 3.29 | - | - | - | - | - | - |
| **U-** | 2.95 | 3.63 | 0.63 | 0.68 | 3.99 | 2.13 | 2.09 | -0.37 | 0.48 | 3.86 | 0.93 | -0.62 | 0.21 | -0.51 | 2.80 | 0.13 | 1.73 | 0.06 | 0.83 | 4.64 | - | - | - | - | - |
| **-A** | 4.14 | 3.35 | 1.48 | 0.71 | 1.21 | 2.48 | 2.35 | -0.55 | -0.43 | -1.43 | 2.26 | -1.02 | 1.28 | 0.33 | 1.67 | 0.38 | 0.54 | 0.24 | 0.44 | -0.11 | 5.15 | - | - | - | - |
| **-C** | 3.53 | 4.88 | 0.63 | 0.67 | 0.36 | 2.85 | 1.95 | -0.34 | 0.31 | 1.31 | 0.83 | 0.35 | 0.63 | 0.71 | 0.14 | 0.14 | 1.73 | -0.26 | 0.73 | 0.21 | 3.63 | 5.61 | - | - | - |
| **-G** | 3.13 | 2.85 | 0.04 | 0.41 | -1.54 | -1.54 | - | -0.58 | -0.70 | 0.31 | -0.07 | -1.16 | 0.50 | -0.29 | 0.24 | -1.30 | -2.77 | 0.23 | 0.15 | -3.54 | 2.11 | 3.32 | 5.77 | - | - |
| **-U** | 3.15 | 2.08 | 0.17 | 0.79 | -0.03 | 1.63 | 2.34 | -0.47 | 0.75 | -0.25 | 0.58 | -1.79 | -0.66 | 0.16 | -2.06 | -0.92 | -0.73 | -1.37 | 0.81 | -0.66 | 3.77 | 3.28 | 2.58 | 5.20 | - |
| **--** | 1.11 | 1.52 | -0.96 | -0.62 | 0.22 | 0.35 | 0.62 | -1.37 | -0.81 | 0.03 | -1.90 | -1.57 | -1.44 | -1.63 | -0.37 | -1.43 | -1.11 | -1.66 | -1.50 | -0.31 | -0.28 | -0.17 | -0.74 | -0.43 | 0.64 |

This extant/extant matrix is symmetrical (with respect to direction of the mutation) and the blank cell represents those mutations which are not observed in the dataset or their expected value is approaching zero during the observed/expected value determination.
