# Supplementary material for: Mutational Patterns in RNA Secondary Structure Evolution Examined in Three RNA Families
Source: PLoS One. 2011 Jun 17;6(6):e20484. doi: 10.1371/journal.pone.0020484 (PMC3117835; doi:10.1371/journal.pone.0020484)
Supplement: Table S5 — Observed/expected base pair substitution matrices combining the mutations among all three RNA families for the ancestral/extant sequence comparison. (DOC) [file pone.0020484.s009.doc]

|  | **AA** | **AC** | **AG** | **AU** | **A-** | **CA** | **CC** | **CG** | **CU** | **C-** | **GA** | **GC** | **GG** | **GU** | **G-** | **UA** | **UC** | **UG** | **UU** | **U-** | **-A** | **-C** | **-G** | **-U** | **--** |
| --- | --- | --- | --- | --- | --- | --- | --- | --- | --- | --- | --- | --- | --- | --- | --- | --- | --- | --- | --- | --- | --- | --- | --- | --- | --- |
| **AA** | 6.07 | 1.98 | 1.30 | 1.01 | - | 0.98 | - | -1.80 | 1.98 | - | 0.98 | -2.22 | 2.08 | -0.56 | - | 0.61 | - | 0.43 | - | -0.51 | 0.89 | 2.08 | - | -0.16 | -4.57 |
| **AC** | 0.98 | 6.05 | - | 1.41 | 2.91 | - | 2.98 | -3.12 | - | 1.24 | 0.24 | 1.13 | - | -0.29 | 1.57 | - | 4.15 | - | - | 1.34 | - | 2.34 | - | - | - |
| **AG** | 1.30 | 3.37 | 6.02 | 0.08 | - | - | 2.30 | -0.80 | 1.57 | -0.44 | -0.44 | -0.18 | 3.08 | 0.61 | - | -0.46 | - | 1.01 | - | - | -1.11 | - | - | - | -5.99 |
| **AU** | 0.86 | 2.20 | 0.08 | 3.87 | -0.02 | -1.05 | 1.01 | -4.09 | 0.76 | -3.05 | -1.73 | -0.39 | -0.95 | 0.54 | - | -1.02 | 1.59 | -1.38 | 0.81 | - | -3.73 | -1.54 | - | -1.60 | -5.43 |
| **A-** | 2.65 | 0.11 | 3.33 | 1.27 | 5.46 | 1.11 | - | -2.67 | 1.69 | 2.28 | - | -4.10 | -0.38 | -0.11 | 1.43 | -1.14 | 2.43 | -0.45 | -0.03 | 1.94 | - | - | - | - | -3.86 |
| **CA** | 2.57 | - | 0.57 | - | - | 6.64 | - | -0.31 | 1.24 | - | 0.24 | -0.64 | - | -0.29 | - | 1.66 | 3.57 | 0.69 | 0.11 | - | - | - | -0.02 | - | -4.31 |
| **CC** | - | 3.57 | 3.30 | - | - | 2.98 | 6.42 | -0.06 | 2.98 | - | - | -0.22 | 1.49 | - | - | 0.74 | 4.89 | -0.16 | - | - | - | 1.49 | - | - | -2.25 |
| **CG** | -1.80 | -1.54 | -0.34 | -0.99 | - | 1.97 | 2.01 | 2.82 | 1.05 | -0.42 | -1.66 | -1.71 | -0.02 | -2.49 | - | -0.86 | 0.79 | 0.91 | -1.67 | -2.61 | -1.99 | -2.61 | -1.22 | -2.67 | -3.61 |
| **CU** | - | - | - | 1.27 | 0.11 | 1.24 | 3.57 | 0.46 | 6.10 | - | - | -0.96 | - | 1.29 | - | -1.59 | 2.57 | - | 2.11 | - | - | - | - | 1.11 | -4.31 |
| **C-** | 2.57 | 0.24 | - | -0.73 | 1.91 | 1.24 | 3.30 | 0.52 | 3.41 | 5.00 | - | -2.96 | 0.76 | -2.29 | 0.98 | -1.00 | - | 0.69 | 0.11 | 1.93 | - | - | - | - | -1.85 |
| **GA** | 3.15 | - | 0.89 | -0.14 | -0.31 | 2.41 | -0.02 | -1.22 | -0.76 | - | 4.33 | -0.96 | 1.57 | 1.03 | -0.44 | 0.87 | 0.57 | -0.31 | -1.90 | -0.66 | 1.15 | -1.24 | -1.02 | 0.11 | -4.14 |
| **GC** | -0.90 | 1.50 | -1.05 | 0.36 | -2.51 | -0.64 | 1.10 | -2.86 | -2.96 | -1.37 | -0.87 | 3.01 | -0.44 | 0.68 | -2.64 | -1.66 | 1.82 | -1.51 | -0.93 | -3.44 | -2.64 | -0.27 | - | - | -4.19 |
| **GG** | - | - | 2.40 | 0.47 | -0.38 | 0.76 | - | 1.04 | 0.76 | - | 0.34 | 0.14 | 4.52 | 1.81 | 1.08 | -0.49 | 2.08 | 1.79 | - | - | 0.08 | - | 0.49 | - | -3.47 |
| **GU** | -0.56 | 1.03 | 0.35 | 1.25 | -2.43 | -1.29 | 1.03 | -2.20 | 2.17 | - | -0.12 | 0.03 | 1.92 | 4.19 | -0.97 | -0.31 | 1.61 | -0.43 | 0.57 | - | - | - | -2.56 | -0.85 | -4.39 |
| **G-** | 0.72 | 0.98 | 1.30 | 0.15 | 1.65 | 0.98 | 0.72 | -0.58 | 1.57 | 1.79 | 1.57 | 0.17 | 1.49 | 1.69 | 4.69 | -0.53 | 2.30 | -1.16 | -1.16 | 0.08 | -1.70 | - | - | - | -2.99 |
| **UA** | 0.74 | -0.26 | -1.26 | -0.59 | -2.72 | 2.27 | -0.26 | -1.66 | -1.59 | -3.59 | -0.68 | -2.33 | -1.07 | -2.31 | -3.85 | 3.33 | 2.91 | 1.41 | 0.86 | -2.07 | -0.36 | -1.49 | -2.85 | -2.72 | -4.55 |
| **UC** | - | 2.57 | 1.89 | 1.86 | - | 2.57 | 4.30 | 0.20 | 4.15 | - | - | 1.17 | - | 0.03 | - | 0.74 | 6.69 | 0.43 | - | - | - | - | - | - | -3.99 |
| **UG** | 2.30 | 0.69 | 2.01 | -0.49 | -0.45 | - | - | 0.13 | - | - | -1.90 | -1.93 | 0.94 | -1.85 | - | 1.03 | 3.75 | 4.39 | 1.43 | -0.06 | -0.57 | -0.38 | -1.16 | -0.45 | -3.86 |
| **UU** | -0.16 | 2.43 | 1.43 | 1.72 | - | 1.69 | 0.84 | -0.67 | 1.69 | 0.69 | 0.43 | -0.77 | -0.38 | 1.57 | -1.16 | 0.45 | 3.43 | 1.55 | 4.70 | 0.21 | - | - | - | 0.55 | -4.86 |
| **U-** | 0.49 | 0.76 | -0.92 | 0.17 | 1.79 | 0.76 | - | -0.70 | -0.24 | 2.46 | -2.24 | -1.12 | -0.73 | 0.03 | 0.82 | 1.25 | 2.66 | 1.21 | 0.21 | 4.50 | - | - | - | - | -2.40 |
| **-A** | 2.47 | 1.15 | - | -1.41 | -1.57 | 1.89 | 1.30 | -2.48 | -0.44 | -1.44 | 0.57 | -1.83 | 0.66 | -0.97 | - | -0.02 | 1.89 | -1.57 | -1.57 | -0.92 | 4.28 | 2.78 | 0.30 | 2.68 | -1.01 |
| **-C** | 0.49 | 2.76 | - | -0.54 | - | 1.76 | - | -0.80 | 0.76 | - | -1.24 | 0.73 | 0.27 | 0.54 | -0.51 | -1.07 | 4.40 | 0.21 | 1.62 | - | 0.08 | 5.23 | 2.49 | 1.62 | -1.55 |
| **-G** | 0.72 | - | 0.89 | -0.51 | - | - | - | -1.39 | - | - | -2.02 | -0.90 | 1.82 | -0.23 | - | -1.53 | 1.30 | 1.30 | -0.16 | - | 0.30 | 0.49 | 4.98 | 1.65 | -0.79 |
| **-U** | -0.16 | - | - | 0.27 | -1.03 | 0.11 | 2.43 | -2.67 | - | - | -0.31 | -2.10 | -0.38 | 1.03 | - | -1.14 | - | - | 0.97 | - | -0.57 | 2.62 | 1.16 | 5.12 | -0.92 |
| **--** | -0.53 | -0.99 | -1.29 | -0.91 | -0.54 | -0.22 | -0.05 | -1.61 | -1.50 | -0.45 | -2.67 | -1.62 | -1.34 | -1.76 | -1.08 | -1.27 | 0.66 | -1.12 | -0.92 | -0.45 | -1.10 | -0.84 | -0.84 | -0.83 | 0.72 |

The blank cell represents those mutations which are not observed in dataset or their expected value is approaching zero during the observed/expected value determination.
