# Supplementary material for: Mutational Patterns in RNA Secondary Structure Evolution Examined in Three RNA Families
Source: PLoS One. 2011 Jun 17;6(6):e20484. doi: 10.1371/journal.pone.0020484 (PMC3117835; doi:10.1371/journal.pone.0020484)
Supplement: Table S6 — The species present in the each cluster in all studied RNA families in PCA. (DOC) [file pone.0020484.s010.doc]

**A**

| **tmRNA species** | **Cluster number** | **Cluster shape in the figure 6A** |
| --- | --- | --- |
| *Helicobacter hepaticus* | 1 | Circle |
| *Acidithiobacillus ferrooxidans* | 1 | Circle |
| *Methylococcus capsulatus* | 1 | Circle |
| *Nitrosococcus oceani* | 1 | Circle |
| *Xanthomonas axonopodis* | 1 | Circle |
| *Xanthomonas oryzae* | 1 | Circle |
| *Xanthomonas campestris* | 1 | Circle |
| *Coxiella burnetii* | 1 | Circle |
| *Chromohalobacter salexigens* | 1 | Circle |
| *Acinetobacter sp. ADP1* | 1 | Circle |
| *Pseudomonas syringae* | 1 | Circle |
| *Pseudomonas fluorescens pf5* | 1 | Circle |
| *Pseudomonas entomophila L48* | 1 | Circle |
| *Pseudomonas aeruginosa PAO1* | 1 | Circle |
| *Azotobacter vinelandii* | 1 | Circle |
| *Idiomarina loihiensis* | 1 | Circle |
| *Shewanella denitrificans* | 1 | Circle |
| *Shewanella frigidimarina* | 1 | Circle |
| *Aeromonas hydrophila 4AK4* | 1 | Circle |
| *Buchnera sp. AJ417833* | 1 | Circle |
| *Buchnera aphidicola Bp* | 1 | Circle |
| *Candidatus Blochmannia* | 1 | Circle |
| *Sodalis glossinidius* | 1 | Circle |
| *Citrobacter rodentium* | 1 | Circle |
| *Salmonella enterica TyphiTy2* | 1 | Circle |
| *Salmonella bongori* | 1 | Circle |
| *Erwinia amylovora* | 1 | Circle |
| *Shigella dysenteriae* | 1 | Circle |
| *Shigella flexneri 301* | 1 | Circle |
| *Shigella boydii* | 1 | Circle |
| *Escherichia coli* | 1 | Circle |
| *Photorhabdus luminescens* | 1 | Circle |
| *Photorhabdus asymbiotica* | 1 | Circle |
| *Yersinia pestis KIM10+* | 1 | Circle |
| *Pasteurella multocida* | 1 | Circle |
| *Haemophilus somnus* | 1 | Circle |
| *Mannheimia succiniciproducens* | 1 | Circle |
| *Haemophilus influenzae* | 1 | Circle |
| *Mannheimia haemolytica* | 1 | Circle |
| *Actinobacillus pleuropneumoniae* | 1 | Circle |
| *Haemophilus ducreyi 35000HP* | 1 | Circle |
| *Vibrio parahaemolyticus* | 1 | Circle |
| *Pseudoalteromonas haloplanktis* | 1 | Circle |
| *Saccharophagus degradans* | 1 | Circle |
| *Francisella tularensis* | 1 | Circle |
| *Desulfovibrio desulfuricans* | 1 | Circle |
| *Desulfotalea psychrophila* | 1 | Circle |
| *Myxococcus xanthus* | 1 | Circle |
| *Syntrophus aciditrophicus* | 1 | Circle |
| *Geobacter sulfurreducens* | 1 | Circle |
| *Geobacter metallireducens* | 1 | Circle |
| *Magnetococcus sp. MC-1* | 1 | Circle |
| *Chlamydia trachomatis* | 1 | Circle |
| *Chlamydophila abortus* | 1 | Circle |
| *Chlamydophila caviae* | 1 | Circle |
| *Mycoplasma pulmonis* | 1 | Circle |
| *Mycoplasma mobile* | 1 | Circle |
| *Mycoplasma penetrans* | 1 | Circle |
| *Actinomyces naeslundii* | 1 | Circle |
| *Bifidobacterium longum* | 1 | Circle |
| *Kineococcus radiotolerans* | 1 | Circle |
| *Klebsiella pneumoniae* | 1 | Circle |
| *Leifsonia xyli* | 1 | Circle |
| *Clavibacter michiganensis* | 1 | Circle |
| *Arthrobacter sp. FB24* | 1 | Circle |
| *Brevibacterium linens* | 1 | Circle |
| *Streptomyces avermitilis* | 1 | Circle |
| *Corynebacterium diphtheriae* | 1 | Circle |
| *Mycobacterium bovis* | 1 | Circle |
| *Mycobacterium smegmatis* | 1 | Circle |
| *Nocardia farcinica* | 1 | Circle |
| *Frankia sp.* | 1 | Circle |
| *Thermobifida fusca* | 1 | Circle |
| *Burkholderia pseudomallei* | 2 | Up triangle |
| *Burkholderia mallei* | 2 | Up triangle |
| *Burkholderia thailandensis* | 2 | Up triangle |
| *Burkholderia sp 383* | 2 | Up triangle |
| *Burkholderia cenocepacia* | 2 | Up triangle |
| *Burkholderia fungorum* | 2 | Up triangle |
| *Burkholderia xenovorans* | 2 | Up triangle |
| *Ralstonia solanacearum* | 2 | Up triangle |
| *Bordetella bronchiseptica* | 2 | Up triangle |
| *Bordetella parapertussis* | 2 | Up triangle |
| *Bordetella pertussis* | 2 | Up triangle |
| *Bordetella avium* | 2 | Up triangle |
| *Comamonas testosteroni* | 2 | Up triangle |
| *Polaromonas sp. JS666* | 2 | Up triangle |
| *Rubrivivax gelatinosus* | 2 | Up triangle |
| *Thiobacillus denitrificans* | 2 | Up triangle |
| *Nitrosospira multiformis* | 2 | Up triangle |
| *Neisseria lactamica* | 2 | Up triangle |
| *Neisseria meningitidis* | 2 | Up triangle |
| *Chromobacterium violaceum* | 2 | Up triangle |
| *Desulfovibrio vulgaris* | 2 | Up triangle |
| *Nostoc punctiforme* | 3 | Plus |
| *Anabaena variabilis* | 3 | Plus |
| *Trichodesmium erythraeum* | 3 | Plus |
| *Thermosynechococcus elongatus* | 3 | Plus |
| *Cyanophora paradoxa* | 4 | Multiply |
| *Odontella sinensis* | 4 | Multiply |
| *Gracilaria tenuistipitata* | 4 | Multiply |
| *Cyanidium caldarium* | 4 | Multiply |
| *Guillardia theta* | 4 | Multiply |
| *Emiliania huxleyi* | 4 | Multiply |
| *Mesostigma viride* | 4 | Multiply |
| *Rhodospirillum rubrum* | 4 | Multiply |
| *Gluconobacter oxydans* | 4 | Multiply |
| *Candidatus Tremblaya* | 4 | Multiply |
| *Mycoplasma hyopneumoniae* | 4 | Multiply |
| *Thermomicrobium roseum* | 5 | Square |
| *Deinococcus geothermalis* | 5 | Square |
| *Chlorobaculum tepidum* | 5 | Square |
| *Bacteroides fragilis* | 5 | Square |
| *Bacteroides thetaiotaomicron* | 5 | Square |
| *Prevotella intermedia* | 5 | Square |
| *Porphyromonas gingivalis* | 5 | Square |
| *Cytophaga hutchinsonii* | 5 | Square |
| *Salinibacter ruber* | 5 | Square |
| *Campylobacter fetus* | 5 | Square |
| *Campylobacter lari* | 5 | Square |
| *Campylobacter jejuni* | 5 | Square |
| *Helicobacter pylori* | 5 | Square |
| *Stenotrophomonas maltophilia* | 5 | Square |
| *Xylella fastidiosa Temecula1* | 5 | Square |
| *Legionella pneumophila* | 5 | Square |
| *Psychrobacter arcticus* | 5 | Square |
| *Psychrobacter cryohalolentis* | 5 | Square |
| *Pseudoalteromonas atlantica* | 5 | Square |
| *Colwellia psychrerythraea* | 5 | Square |
| *Shewanella amazonensis* | 5 | Square |
| *Wigglesworthia glossinidia* | 5 | Square |
| *Vibrio cholerae* | 5 | Square |
| *Vibrio vulnificus* | 5 | Square |
| *Photobacterium profundum* | 5 | Square |
| *Thiomicrospira crunogena* | 5 | Square |
| *Desulfuromonas acetoxidans* | 5 | Square |
| *Bacteriovorax marinus* | 5 | Square |
| *Gemmata obscuriglobus* | 5 | Square |
| *Dehalococcoides ethenogenes* | 5 | Square |
| *Borrelia garinii* | 5 | Square |
| *Treponema denticola* | 5 | Square |
| *Leptospira interrogans* | 5 | Square |
| *Solibacter usitatus* | 5 | Square |
| *Fibrobacter succinogenes* | 5 | Square |
| *Verrucomicrobium spinosum* | 5 | Square |
| *Bacillus licheniformis* | 5 | Square |
| *Bacillus halodurans* | 5 | Square |
| *Bacillus clausii* | 5 | Square |
| *Paenibacillus larvae* | 5 | Square |
| *Bacillus thuringiensis* | 5 | Square |
| *Bacillus anthracis* | 5 | Square |
| *Bacillus cereus* | 5 | Square |
| *Oceanobacillus iheyensis* | 5 | Square |
| *Listeria monocytogenes* | 5 | Square |
| *Staphylococcus aureus Mu50* | 5 | Square |
| *Lactobacillus acidophilus* | 5 | Square |
| *Lactobacillus gasseri* | 5 | Square |
| *Lactobacillus brevis* | 5 | Square |
| *Pediococcus pentosaceus* | 5 | Square |
| *Lactobacillus casei* | 5 | Square |
| *Streptococcus suis* | 5 | Square |
| *Streptococcus uberis* | 5 | Square |
| *Streptococcus equi* | 5 | Square |
| *Streptococcus thermophilus* | 5 | Square |
| *Streptococcus gordonii* | 5 | Square |
| *Streptococcus pneumoniae* | 5 | Square |
| *Leuconostoc mesenteroides* | 5 | Square |
| *Oenococcus oeni* | 5 | Square |
| *Mycoplasma capricolum* | 5 | Square |
| *Mycoplasma genitalium* | 5 | Square |
| *Mycoplasma gallisepticum* | 5 | Square |
| *Onion yellows* | 5 | Square |
| *Aster yellows* | 5 | Square |
| *Mycoplasma mycoides* | 5 | Square |
| *Spiroplasma kunkelii* | 5 | Square |
| *Clostridium perfringens* | 5 | Square |
| *Clostridium acetobutylicum* | 5 | Square |
| *Clostridium tetani* | 5 | Square |
| *Clostridium difficile* | 5 | Square |
| *Ruminococcus albus* | 5 | Square |
| *Fusobacterium nucleatum* | 5 | Square |
| *Desulfitobacterium hafniense* | 5 | Square |
| *Carboxydothermus hydrogenoformans* | 5 | Square |
| *Moorella thermoacetica* | 5 | Square |
| *Rubrobacter xylanophilus* | 5 | Square |
| *Corynebacterium efficiens* | 5 | Square |
| *Corynebacterium glutamicum* | 5 | Square |
| *Coprothermobacter proteolyticus* | 5 | Square |

B

| ***RNaseP A species*** | **Cluster number** | **Cluster shape in the fig. S4A** |
| --- | --- | --- |
| *Rickettsia prowazekii* | 1 | Circle |
| *Wolbachia sp.* | 1 | Circle |
| *Nitrosomonas europaea* | 1 | Circle |
| *Neisseria meningitidis* | 1 | Circle |
| *Acidimicrobium ferrooxidans* | 1 | Circle |
| *Buchnera aphidicola* | 1 | Circle |
| *Chromatium vinosum* | 1 | Circle |
| *Pseudomonas fluorescens* | 1 | Circle |
| *Pseudomonas aeruginosa* | 1 | Circle |
| *Xylella fastidiosa* | 1 | Circle |
| *Shewanella putrefaciens* | 1 | Circle |
| *Desulfovibrio vulgaris* | 1 | Circle |
| *Campylobacter jejuni* | 1 | Circle |
| *Helicobacter pylori* | 1 | Circle |
| *Clostridium acetobutylicum* | 1 | Circle |
| *Bacteroides thetaiotaomicron* | 1 | Circle |
| *Borrelia burgdorferi* | 1 | Circle |
| *Treponema pallidum* | 1 | Circle |
| *Thermus aquaticus* | 1 | Circle |
| *Thermus thermophilus* | 1 | Circle |
| *Thermotoga maritima* | 1 | Circle |
| *Thermotoga neapolitana* | 1 | Circle |
| *Rhodobacter capsulatus* | 2 | Up triangle |
| *Escherichia coli* | 2 | Up triangle |
| *Enterobacter agglomerulans* | 2 | Up triangle |
| *Haemophilus influenzae* | 2 | Up triangle |
| *Klebsiella pneumoniae* | 2 | Up triangle |
| *Salmonella typhi* | 2 | Up triangle |
| *Serratia marcescens* | 2 | Up triangle |
| *Vibrio cholerae* | 2 | Up triangle |
| *Yersinia pestis* | 2 | Up triangle |
| *Pseudanabaena sp PCC6903* | 2 | Up triangle |
| *Caulobacter crescentus* | 3 | Plus |
| *Rhodospirillum rubrum* | 3 | Plus |
| *Alcaligenes eutrophus* | 3 | Plus |
| *Desulfovibrio desulfuricans* | 3 | Plus |
| *Geobacter sulfurreducens* | 3 | Plus |
| *Corynebacterium diphtheriae* | 3 | Plus |
| *Mycobacterium avium* | 3 | Plus |
| *Mycobacterium bovis* | 3 | Plus |
| *Mycobacterium leprae* | 3 | Plus |
| *Mycobacterium tuberculosis* | 3 | Plus |
| *Streptomyces bikiniensis* | 3 | Plus |
| *Streptomyces lividans* | 3 | Plus |
| *Aspergillus nidulans* | 3 | Plus |
| *Prochlorococcus marinus* | 3 | Plus |
| *Porphyromonas gingivalis* | 3 | Plus |
| *Chlamydophila abortus* | 3 | Plus |
| *Chlamydophila caviae* | 3 | Plus |
| *Chlamydophila felis* | 3 | Plus |
| *Chlamydia trachomatis* | 3 | Plus |
| *Chlamydia muridarum* | 3 | Plus |
| *Chlamydophila pneumoniae* | 3 | Plus |
| *Chlamydia suis* | 3 | Plus |
| *Chlorobium limicola* | 3 | Plus |
| *Chlorobaculum tepidum* | 3 | Plus |
| *Deinococcus radiodurans* | 3 | Plus |
| *Rhodopseudomonas palustris* | 4 | Multiply |
| *Bordetella pertussis* | 4 | Multiply |
| *Calothrix sp. PCC7601* | 4 | Multiply |
| *Prochlorothrix hollandica* | 4 | Multiply |
| *Synechocystis sp. PCC6803* | 4 | Multiply |

**C**

| **RNaseP B** | **Cluster number** | **Cluster shape in the fig. S4B** |
| --- | --- | --- |
| *Heliobacillus mobilis* | 1 | Circle |
| *Heliobacterium chlorum* | 1 | Circle |
| *Lactobacillus acidophilus* | 1 | Circle |
| *Streptococcus equinus* | 1 | Circle |
| *Streptococcus faecium* | 1 | Circle |
| *Erysipelothrix rhusiopathiae* | 1 | Circle |
| *Acholeplasma laidlawii* | 1 | Circle |
| *Clostridium innocuum* | 1 | Circle |
| *Enterococcus faecalis* | 1 | Circle |
| *Bacillus subtilis* | 2 | Up triangle |
| *Bacillus halodurans* | 2 | Up triangle |
| *Bacillus megaterium* | 2 | Up triangle |
| *Staphylococcus aureus* | 2 | Up triangle |
| *Staphylococcus epidermidis* | 2 | Up triangle |
| *Streptococcus mutans* | 2 | Up triangle |
| *Streptococcus gordonii* | 2 | Up triangle |
| *Streptococcus pneumoniae* | 2 | Up triangle |
| *Streptococcus pyogenes* | 2 | Up triangle |
| *Mycoplasma capricolum* | 2 | Up triangle |
| *Mycoplasma fermentans* | 2 | Up triangle |
| *Bacillus anthracis* | 2 | Up triangle |
| *Bacillus stearothermophilus* | 2 | Up triangle |
| *Bacillus brevis* | 2 | Up triangle |
| *Mycoplasma flocculare* | 3 | Plus |
| *Mycoplasma hyopneumoniae* | 3 | Plus |

**D**

| **Vertebrate telomerase RNA** | **Cluster number** | **Cluster shape in the fig S4C** |
| --- | --- | --- |
| *Microtus ochrogaster* | 1 | Circle |
| *Cricetulus griseus* | 1 | Circle |
| *Mus spretus* | 1 | Circle |
| *Mus musculus* | 1 | Circle |
| *Rattus norvegicus* | 1 | Circle |
| *Elephas maximus* | 2 | Up triangle |
| *Trichechus manatus* | 2 | Up triangle |
| *Oryctolagus cuniculus* | 2 | Up triangle |
| *Cavia porcellus* | 2 | Up triangle |
| *Chinchilla brevicaudata* | 2 | Up triangle |
| *Geomys breviceps* | 2 | Up triangle |
| *Homo sapiens* | 2 | Up triangle |
| *Tupaia glis belangeri* | 2 | Up triangle |
| *Mustela putorius furo* | 2 | Up triangle |
| *Procyon lotor* | 2 | Up triangle |
| *Felis catus* | 2 | Up triangle |
| *Bos taurus* | 2 | Up triangle |
| *Sus scrofa* | 2 | Up triangle |
| *Suncus murinus* | 2 | Up triangle |
| *Equus caballus* | 2 | Up triangle |
| *Dasypus novemcinctus* | 2 | Up triangle |
| *Rhizoprionodon porosus* | 3 | Plus |
| *Mustelus canis* | 3 | Plus |
| *Dasyatis sabina* | 3 | Plus |
| *Rhinoptera bonasus* | 3 | Plus |
| *Xenopus laevis* | 3 | Plus |
| *Bufo japonicus* | 3 | Plus |
| *Ceratophrys ornata* | 3 | Plus |
| *Pyxicephalus adspersus* | 3 | Plus |
| *Dermophis mexicanus* | 3 | Plus |
| *Herpele squalostoma* | 3 | Plus |
| *Typhlonectes natans* | 3 | Plus |
| *Gallus gallus* | 3 | Plus |
| *Anodorhynchus hyacinthinus* | 3 | Plus |
| *Chelydra serpentina* | 3 | Plus |

**E**

| **Ciliate telomerase RNA** | **Cluster number** | **Cluster shape in the figure 6B** |
| --- | --- | --- |
| *Colpidium colpoda* | 1 | Circle |
| *Colpidium campylum* | 1 | Circle |
| *Glaucoma chattoni* | 1 | Circle |
| *Tetrahymena silvana* | 1 | Circle |
| *Tetrahymena vorax* | 1 | Circle |
| *Tetrahymena borealis* | 1 | Circle |
| *Tetrahymena thermophila* | 1 | Circle |
| *Tetrahymena capricornis* | 1 | Circle |
| *Tetrahymena australis* | 1 | Circle |
| *Tetrahymena paravorax* | 1 | Circle |
| *Colpidium striatum* | 1 | Circle |
| *Euplotes aediculatus* | 2 | Up triangle |
| *Euplotes eurystomus* | 2 | Up triangle |
| *Moneuplotes crassus* | 2 | Up triangle |
| *Oxytricha nova* | 2 | Up triangle |
| *Oxytricha trifallax* | 2 | Up triangle |
| *Stylonychia mytilis* | 2 | Up triangle |
| *Stylonychia lemnae* | 2 | Up triangle |
| *Paramecium primaurelia* | 3 | Plus |
| *Paramecium tetraurelia* | 3 | Plus |
| *Paramecium multimicronucleatum* | 3 | Plus |
| *Paramecium caudatum* | 3 | Plus |
